# Modulation of apoptosis in human hepatocellular carcinoma (HepG2 cells) by a standardized herbal decoction of *Nigella sativa *seeds, *Hemidesmus indicus *roots and *Smilax glabra *rhizomes with anti- hepatocarcinogenic effects

**DOI:** 10.1186/1472-6882-12-25

**Published:** 2012-03-29

**Authors:** Sameera R Samarakoon, Ira Thabrew, Prasanna B Galhena, Kamani H Tennekoon

**Affiliations:** 1 Institute of Biochemistry, Molecular Biology and Biotechnology, University of Colombo, 90, Cumarathunga Munidasa Mawatha, Colombo 3, Sri Lanka; 2 Department of Biochemistry and Clinical Chemistry, Faculty of Medicine, University of Kelaniya, Thalagolla Road, Ragama, Sri Lanka

## Abstract

**Background:**

A standardized poly-herbal decoction of *Nigella sativa *seeds, *Hemidesmus indicus *roots and *Smilax glabra *rhizomes used traditionally in Sri Lanka for cancer therapy has been demonstrated previously, to have anti-hepatocarcinogenic potential. Cytotoxicity, antioxidant activity, anti-inflammatory activity, and up regulation of p53 and p21 activities are considered to be some of the possible mechanisms through which the above decoction may mediate its anti-hepatocarcinogenic action. The main aim of the present study was to determine whether apoptosis is also a major mechanism by which the decoction mediates its anti-hepatocarcinogenic action.

**Methods:**

Evaluation of apoptosis in HepG2 cells was carried out by (a) microscopic observations of cell morphology, (b) DNA fragmentation analysis, (c) activities of caspase 3 and 9, as well as by (d) analysis of the expression of pro-apoptotic (Bax) and anti-apoptotic (Bcl-2) proteins associated with cell death.

**Results:**

The results demonstrated that in HepG2 cells, the decoction can induce (a) DNA fragmentation and (b) characteristic morphological changes associated with apoptosis (nuclear condensation, membrane blebbing, nuclear fragmentation and apoptotic bodies). The decoction could also, in a time and dose dependent manner, up regulate the expression of the pro-apoptotic gene *Bax *and down regulate expression of anti-apoptotic *Bcl-2 *gene (as evident from RT-PCR analysis, immunohistochemistry and western blotting). Further, the decoction significantly (*p *< .001) enhanced the activities of caspase-3 and caspase-9 in a time and dose dependent manner.

**Conclusions:**

Overall findings provide confirmatory evidence to demonstrate that the decoction may mediate its reported anti-hepatocarcinogenic effect, at least in part, through modulation of apoptosis.

## Background

A decoction prepared from a mixture of *Nigella sativa *seeds, *Hemidesmus indicus *roots and *Smilax glabra *rhizomes has traditionally been used for many years by a particular family of Ayurveda physicians in Sri Lanka [1] for the treatment of cancer patients. Findings of recent in vivo investigations carried out by Iddamaldeniya *et al.*, (2003 and 2006) indicate that this poly-herbal decoction has the potential to significantly inhibit chemically induced hepatocarcinogenesis [1,2]. The decoction has been standardized considering its High Performance Liquid Chromatography (HPLC) profile and physico-chemical parameters according to WHO guidelines [3]. Further in vivo and in vitro investigations have demonstrated that cytotoxicity, [3,4] antioxidant activity, anti-inflammatory activity [5] and up regulation of p53 and p21 activities [6] are some of the possible mechanisms through which the above decoction may mediate its anti-hepatocarcinogenic action.

Apoptosis or programmed cell death plays a crucial role in maintaining cellular homeostasis between cell division and cell death [7,8]. An imbalance between cell proliferation and apoptotic cell death will result in serious diseases such as cancer [9]. Cell death mediated by apoptosis results in a series of morphological changes such as nuclear condensation, nuclear fragmentation, and cell surface blebbing, which leads to the formation of membrane bound vesicles (apoptotic bodies) being subsequently phagocytised by macrophages [10]. Apoptosis is regarded as the most preferred way to manage cancer as this process does not affect neighbouring cells like in necrosis. Apoptosis can occur through a death receptor (extrinsic) pathway or a mitochondrial (intrinsic) pathway [11,12]. Both pathways will result in the activation of caspases, a family of enzymes that act as death effector molecules in various forms of cell death [13]. Apoptosis has been demonstrated to be a major mechanism employed by many natural agents [14,15] to mediate anticancer effects.

Hepatocellular carcinoma (HCC) is one of the commonest malignant diseases of the world, and the most frequent primary liver cancer, with continuously increasing incidence over the past decade [16]. Since the decoction comprised of *Nigella sativa *seeds, *Hemidesmus indicus *roots and *Smilax glabra *rhizomes has been demonstrated experimentally to possess anti-hepatocarcinogenic properties [1,2], for further development of this decoction for the treatment of human HCC, it is important to investigate whether apoptosis is also a major mechanism by which it can mediate anticarcinogenic effects. Results of a preliminary flow cytometric analysis carried out recently by Thabrew *et al.*, (2005) with HepG2 cells, indicated that the above decoction may have the potential to induce apoptosis [4]. Therefore the present study was carried out with the aim of obtaining further evidence to confirm that apoptosis is indeed a major mechanism through which the test decoction mediates its anti-hepatocarcinogenic effects. In the present study, evaluation of apoptosis in HepG2 cells was carried out by (a) microscopic observations of cell morphology, (b) DNA fragmentation analysis, (c) activities of caspase 3 and 9, as well as by (d) analysis of the expression of pro-apoptotic (Bax) and anti-apoptotic (Bcl-2) proteins associated with cell death.

## Methods

### Materials and methods

HepG2 cells were purchased from ECACC, Salisbury, UK. Dulbecco's Modified Eagle Medium (DMEM), and TRIzol reagent were purchased from Invitrogen, Life Technologies; U.S.A. Fetal bovine serum (FBS), streptomycin/penicillin, dimethyl sulfoxide (DMSO), agarose, Bradford's reagent and trypsin/EDTA and all other chemicals were purchased from Sigma Aldrich (Gillingham, Dorset, UK). M-MLV reverse transcriptase system (A3500), and GoTaq DNA polymerase, were purchased from Promega Cooperation, Madisons, U.S.A. PCR primers were purchased from Integrated DNA Technologies (IDT) U.S.A. Primary and secondary antibodies, ABC staining kit and western blotting luminol reagent were purchased from Santa Cruz Biotechnology (Santa Cruz, CA). Caspase-3 and caspase-9 colorimetric kit was purchased from GenScript USA.

### Collection of plant material

Plant material for the preparation of extracts and their authentication has been previously reported (Samarakoon et al., 2010). Plant material was purchased from a reputed vendor of herbal material used by traditional medical practitioners in Sri Lanka (D. J. Fernando Pvt Ltd, Gabose lane, Colombo 13). Voucher specimens of *N. sativa *seeds, *H. indicus *roots, and *S.glabra *rhizome (Voucher specimen nos.UOC/IBMBB/2009/01, UOC/IBMBB/2009/02 and UOC/IBMBB/2009/03), have been deposited at the Institute of Biochemistry, Molecular Biology & Biotechnology, University of Colombo, Sri Lanka.

### Preparation of plant decoction

The plant decoction was prepared as described previously (Samarakoon *et al.*, 2010). Sixty grams (60 g) of plant material (composed of a mixture of 20 g each of *N. sativa *seeds, *H. indicus *roots and *S. glabra *rhizomes) were ground and boiled gently with 1.6 L distilled water for approximately 3 h to reduce the volume to 200 ml. The extract was then filtered through a layer of muslin, filtrate centrifuged at 3000 g for 15 min to remove any debris, and the supernatant freeze dried and stored at -20°C until required. When required for experimental purposes, the freeze dried extract was reconstituted with the appropriate volume of distilled water containing 1% DMSO.

### Cell culture

HepG2 cells were grown in monolayer cultures in Dulbecco's Modified Eagle Medium (DMEM) supplemented with 10% fetal bovine serum, and 50 IU/ml penicillin and 50 μg/ml streptomycin. Cells were maintained at 37°C in 95% air/5% CO_2 _atmosphere, with 95% humidity.

### Evaluation of HepG2 cell viability by MTT assay

The viability of HepG2 cells (2 × 10^5 ^cells/ml) incubated with the decoction for 24 h with different doses of the decoction were evaluated by the MTT (3-(4,5-Dimethylthiazol-2-yl)-2,5-diphenyltetrazolium bromide) assay as described previously [3].

### Effects of the decoction on viability of peripheral blood mononuclear cells

The peripheral blood mononuclear cells collected from blood of healthy volunteers was used to evaluate effects of the decoction on normal (non-cancerous) human cells. Mononuclear cells were isolated by separation in Histopaque 1077 (Sigma). Diluted blood with Hank's Buffered Salt Solution (HBSS) was carefully layered onto Histopaque 1077 and centrifuged at 1000 rpm for 10 min. After centrifugation the opaque interface containing mononuclear cells were collected and washed with phosphate buffered saline (PBS). After isolation, cells in RPMI medium with 50 U/ml penicillin, 50 μg/ml streptomycin and 10% fetal bovine serum (Sigma) were seeded at a cell density of 2 × 10^5 ^cells/ml in 24 well plates. Cells were maintained at 37°C in 95% air/5% CO_2 _atmosphere, with 95% humidity. Cultures were exposed only to medium containing 1% DMSO (controls) or medium containing different concentrations of decoction dissolved in 1% DMSO (600 μg/ml -2400 μg/ml), and incubated for 24 h. At the end of this incubation period, to determine percentage of viable cells, the above cells were collected by centrifugation at 150 rpm for 10 min and resuspended in 0.5 ml of RPMI medium. Cells (100 μl) were then mixed with 1:1 dilution of (100 μl) 0.4% trypan blue solution. Cell were counted using hemocytometer.

### Isolation of total RNA and RT-PCR analysis of bax and bcl-2 genes in HepG2 cells

HepG2 cells cultured for 24 h were incubated for 12, 24 and 48 h respectively, with fresh medium containing different concentrations of the decoction (test cells) or 1% DMSO (control cells). The concentrations of decoction used were 75, 150, 300, 600, and 1200 μg/ml, and triplicate cell cultures were exposed to each concentration. Treated and control cell cultures were incubated for a further 12, 24 or 48 h. At the end of the incubation period cells were harvested and used for total RNA extraction for reverse transcription PCR (RT- PCR).

Total RNA was isolated from the HepG2 cells, using TRIzol reagent according to the manufacturer's specifications. Total RNA concentration in the final elutes was determined by using a spectrophotometer (UV-1700, pharmasprc, SHIMADZU, Japan). Each sample of isolated RNA was reverse transcribed by M-MLV reverse transcriptase system.

Each PCR was carried out in a master mix containing 1X Green Go Taq Flexi Buffer, 2 mM MgCl_2_, 10 mM dNTPs and 1.25 U GoTaq DNA polymerase (Promega Inc. US) with 0.2 mM of respective forward and reverse primers in 50 μl reaction mix. The PCR amplification was carried out in a Mastercycler (Eppendrof, Hamburg, Germany). *GAPDH *(glyceraldehydes 3-phosphate dehydrogenase) amplification was performed as an internal control. PCR conditions for *GAPDH *were 30 cycles at 94°C for 30 sec, at 54°C for 30 sec at 72°C for 1 min. PCR conditions for *Bax *were 35 cycles at 94°C for 1.5 min, at 55°C for 30 sec, at 72°C for 1 min. PCR conditions for *Bcl-2 *were 30 cycles at 94°C for 1.5 min, at 56°C for 30 sec at72°C for 1 min. The primers used for RT-PCR are shown in Table 1. Amplified PCR products were subjected to electrophoresis at 40 V through 2% agarose gel for 60 min. A 100 bp DNA ladder marker was used as a molecular marker. Gels were stained with 0.5 mg/ml ethidium bromide in TAE (Tris-acetate-EDTA) buffer. The gel bands were examined by using a Gel Doc imaging system and the intensity of each band was measured by using BIO RAD quantity one soft ware. Size of the amplified PCR products were 150 bp for Bax, 129 bp for Bcl-2, and 226 bp for *GAPDH*.

**Table 1:** Primer sequences used for RT PCR

Name of Primer	Primer sequence
*Bax *: forward primer	5'-GGACGAACTGGACAGTAACATGG-3'
*Bax *: reverse primer	5'-GCAAAGTAGAAAAGGGCGACAAC-3'
*Bcl-2*: forward primer	5'-ATCGCCCTGTGGATGACTGAG-3'
*Bcl-2*: reverse primer	5'- CAGCCAGGAGAAATCAAACAGAGG-3'
*GAPDH*: forward primer	5'-GAAGGTGAAGGTCGGAGTC-3'
*GAPDH*: reverse primer	5'-GAAGATGGTGATGGGATTTC-3'

### Immunohistochemistry

HepG2 cells harvested by trypsinization, and 2 × 10^5 ^cells/ml were plated on sterile coverslips placed in wells of a 24 well cell culture plate. The cells (on coverslips) were initially maintained in DMEM medium (1 ml/coverslip) for 24 h at 37°C in 95% air/5% CO_2 _atmosphere, with 95% humidity. Cultures were then treated with the standardized decoction (600 μg/ml or 1200 μg/ml) and incubated for 24 h. Cells were then washed with phosphate buffer saline (PBS), fixed with cold 1:1 acetone: methanol for 15 minutes and then air dried. The coverslips with HepG2 cells were quenched with 1% BSA in 0.05 M Tris/HCl (pH 7.6) for 30 min, and then incubated for 60 min at 37°C with anti-Bax and anti-BCL-2 rabbit polyclonal antibodies, diluted 1: 200. After washing with PBS (two times), the cells were incubated for 60 min with goat anti-rabbit IgG-HRP (diluted 1: 1000), washed again with PBS (two times) and visualized with ABC staining kit. Cells were counterstained with hematoxylin and examined under a light microscope.

### Western blot analysis

For *in vitro *evaluation of Bax and Bcl-2 proteins in HepG2 cells by western blot analysis, cells were harvested after 12, 24 and 48 h incubation with the decoction and centrifuged at 1000 g for 10 min at 4°C. The pelleted cells were solubilized for 15 min at 4°C in lysis buffer [50 mM Tris-HCl pH 7.4, 150 mM NaCl, 1 mM EDTA, 1% Nonidet P-40, 0.25% Na-deoxycholate, 1 mM PMSF (phenylmethylsulfonyl fluoride) and proteinase inhibitor cocktail]. Lysates were centrifuged at 12,000*g *for 15 min at 4°C to remove insoluble material. The supernatant was collected and protein concentration was determined using the Bradford's reagent. For Western blot analysis, samples (40 μg each) were separated by electrophoresis on 10% SDS-polyacrylamide gels and transferred onto nitrocellulose membranes. The nitrocellulose membranes onto which proteins were transferred were pre-blocked with 5% non fat dry milk in PBS for 30 min and then incubated for 16 h at 4°C with specific primary antibodies. After three washes of 10 min each with 1× TBST (50 mM Tris-HCl, pH7.4, 150 mM NaCl, 1% Tween 20), each membrane was incubated with the secondary antibody for 30 min at room temperature. The membrane was then washed three times with TBST (10 min/wash) and bands visualized using western blotting luminol reagent system. Bands were normalized using β-actin antibody.

### Detection of morphological changes related to apoptosis by light microscopy

HepG2 cells (2 × 10^5 ^cells/ml) cultured in 24 well cell culture plates were exposed to different concentrations (0-1800 μg/ml) of the decoction (test cells) or 1% DMSO (control cells) respectively for further 24 h. Morphological changes of the cells were then observed under a inverted light microscope (Olympus CKX41SF, Japan).

#### Detection of morphological changes related to apoptosis by fluorescent microscopy

HepG2 cells were seeded at a final concentration of 2 × 10^5 ^cells/ml in 6-well culture plates the night before the treatment for apoptosis detection by acridine orange/ethidium bromide (AO/EB) staining and Hoechst 33258 staining. The cells were exposed to different concentrations of the decoction (0-1800 μg/ml) (test cells) or 1% DMSO (control cells) respectively for 24 h. After incubation, control and treated cells were harvested by trypsinization, centrifuged and suspended in PBS at a final concentration of 1 × 10^5 ^cells/ml. The cells were fixed by 4% formaldehyde at room temperature and plated onto glass slides and subjected to apoptosis analysis by acridine orange/ethidium bromide (AO/EB) staining and Hoechst 33258 staining as described below.

#### Acridine orange/ethidium bromide (AO/EB) staining

The AO/EB staining was carried out according to Ribble et al. (2005) [17]. Fluorescent dyes, ethidium bromide (100 μg/ml) and acridine orange (100 μg/ml) were added to the fixed cells and incubated for 10 min at room temperature in dark. Changes in the nuclei of cells within 15 min. after AO/EB staining were observed under a fluorescence microscope (Olympus, BX51TRF, Japan). Morphological criteria were used to evaluate cell injury. Cells containing normal nuclear chromatin exhibit green nuclear staining. Cells containing fragmented nuclear chromatin due to apoptosis exhibit orange to red nuclear staining.

#### Hoechst 33258 staining

Characteristic apoptotic morphological changes were assessed by fluorescent microscopy using bis-benzimide (Hoechst 33258) staining. Fixed cells were stained with 200 μL of bis-benzimide (5 μg/mL) for 10 min at room temperature. Then, 10 μL of this suspension was placed on a glass slide and covered with a cover slip. The cells were examined using a fluorescence microscope (Olympus BX51TRF, Japan), to determine nuclei fragmentation and chromatin condensation.

### DNA fragmentation analysis

Agarasose gel electrophoresis was used to detect the characteristic ladder pattern of DNA fragmentation as described by Yang et al. (2000) with slight modifications. Cells (2 × 10^5 ^cells/ml) exposed to the standardized decoction and positive control (thymoquinone) for 24 and 48 h were gently scraped and harvested by centrifugation. The cell pellets were incubated for 60 min at 50°C in 100 μl lysis buffer (100 mM Tris-HCl pH 8, 100 mM NaCl and 10 mM EDTA). Proteinase K (10 μl 20 mg/ml stock solution) was then added to the lysis mixture and further incubated for 30 min at 50°C. RNase (3 μl from 10 mg/ml stock solution) was then added and the mixture incubated for 2 h at 50°C. DNA was extracted with phenol-chloroform-isoamyl alcohol, subjected to 2.0% of agarose gel electrophoresis, stained with ethidium bromide and visualized under UV light using a gel-doc system (Quantum-ST4 1100/20 M).

### Caspase 3 and caspase 9 activities

Caspase activities were determined by colorimetric assays using caspase-3, and -9 activation kits (GenScript USA) according to the manufacturer's protocol. The kits are based on spectrophotometric detection of the chromophore *p*nitroaniline (*p*NA) after cleavage from the labeled substrate DEVD-*p*NA. HepG2 cells (2 × 10^5 ^cells/ml) were incubated with different concentrations of the decoction (600 μg/ml and 1200 μg/ml) for 24, and 48 h. Cells were then harvested by trypsinization, washed twice with ice-cold PBS buffer (pH 7.4) and lysed in the supplied lysis buffer containing dithiothreitol (DTT). The supernatant was collected and protein concentration determined using Bradford's reagent. Equal amount of protein (100 μg) was incubated with the supplied reaction buffer containing dithiothreitol (DTT) and substrates (5 μl) at 37°C for 4 h. The caspase activity was determined by measuring changes in absorbance at 405 nm using the Micro plate reader (EL_x _800 Universal Microplate Reader, BIO-TEK INSTRUMENTS, USA).

### Statistical analysis

Results of RT-PCR were analyzed using Prism 2.01 (Graphpad Prism, San Diego, CA). Two way ANOVA was used to detect the effect of concentrations of the decoction and duration of the treatment on the mRNA expression of *Bax *and *Bcl-2 *in HepG2 cells. One way analysis of variance (One way ANOVA) with Dunnett's post test for multiple comparisons was used to find out the concentrations at which the *Bax *expression was significantly increased while reducing the expression of Bcl-2 in comparison to controls. Effect of the decoction on caspase activities was analysed by using one way ANOVA with Banforroni post test.

## Results

### RT-PCR analysis of bax and bcl-2 gene expression in HepG2 cells

**Figure 1:**
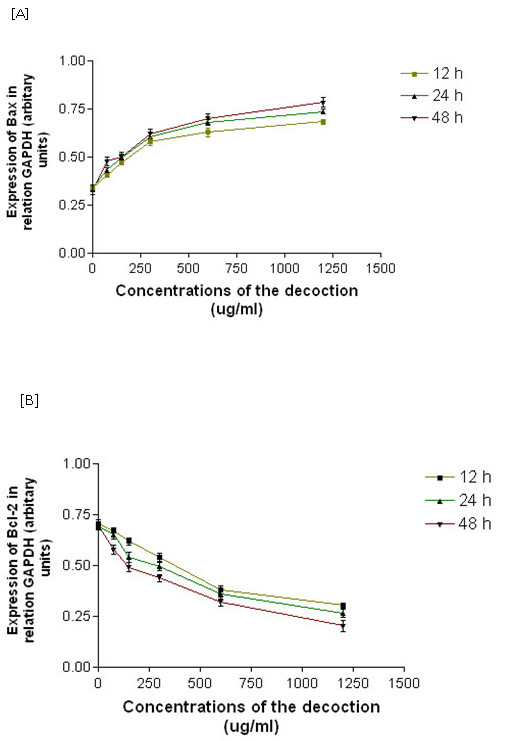
**Effects of the decoction on mRNA expression of Bax and Bcl-2 in HepG2 cells treated with the decoction for 12, 24, and 48 h**. Data values are expressed as mean ± SEM of triplicate determinations. Significant differences were compared to the control at p* <.01 by ANOVA with Dunnett's post test for different time points and Two way ANOVA analysis was used to detect the effect of different concentrations of the decoction and duration of the treatment on the mRNA expression of *Bax *and *Bcl-2 *in HepG2 cells.

*Bax *and *Bcl-2 *gene expression were normalized to the house keeping *GAPDH *gene. As evident from Figure 1A, B, Additional file: Figure S1, and Table 2, RT-PCR evaluation of HepG2 cells treated with different concentrations of the decoction (75 μg/ml - 1200 μg/ml) showed in a significant (*P *< 0.001) dose and time dependent increase in *Bax *mRNA expression along with a significant (*P *< 0.001) decrease in *Bcl-2 *mRNA expression.

**Table 2:** Bax/Bcl-2 ratio of mRNA expression

Dose of the decotion	Bax/Bcl-2		
	
	12 h	24 h	48 h
Control	0.484	0.489	0.468
75 μg/ml	0.603	0.663	0.827
150 μg/ml	0.758	0.920	1.020
300 μg/ml	1.074	1.220	1.410
600 μg/ml	1.660	1.886	2.180
1200 μg/ml	2.254	2.792	3.857

Bax/Bcl-2 ratio was up regulated in a time and dose dependent manner

### Immunohistochemical analysis and western blot analysis of bax and bcl-2 in HepG2 cells

**Figure 2:**
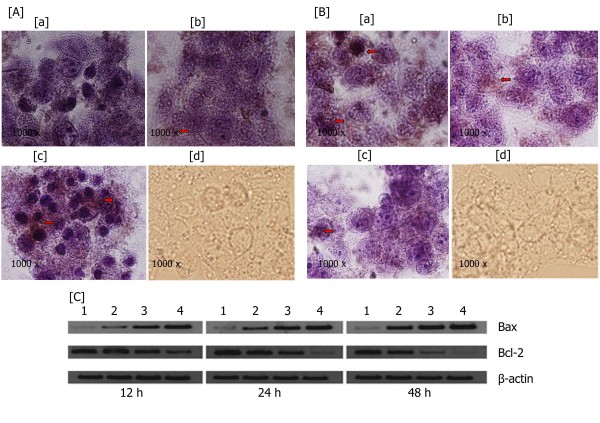
**Immunohistochemistry and Western blot analysis**. Immunohistochemical analysis analysis of expression of Bax [A] and Bcl-2 [B] protein in HepG2 cells (shown by arrows). [a] control (untreated) [b] treated with the decoction (600 μg/ml), [c] treated with the decoction (1200 μg/ml). Section [d] represents the negative control. Hematoxyline was used for counterstaining. [C] Western blot analysis of effects of the decoction on Bax and Bcl-2 expression in HepG2 cells treated with different concentrations (lane 1-control, lane 2-300 μg/ml, lane 3-600 μg/ml, lane 4-1200 μg/ml) of the standardized decoction at (a) 12 h post incubation, (b) 24 h post-incubation and (c) 48 h post incubation was performed as described in the Materials and Methods section. Normalization of bands was performed using β-actin protein.

As evident from Figure 2A and 2B, immunohistochemical analysis of Bax and Bcl-2 proteins confirmed the results from RT-PCR analysis of *Bax *and *Bcl-2 *mRNA expression in HepG2 cells. Expression of Bax was increased and Bcl-2 was decreased in treated samples when compared to control samples. In western blot analysis *Bax *and *Bcl-2 *gene expression were normalized to the house keeping β-actin protein. In support of results obtained by immunohistochemistry and RT-PCR, results of the western blot analysis of Bax and Bcl-2 expression in HepG2 cells (2 × 10^5 ^cells/ml) treated with different concentrations (0-1200 μg/ml) of the standardized decoction (for 12, 24 and 48 h) also resulted in a time and dose dependent up regulation of Bax along with a down regulation of Bcl-2 (Figure 2C.).

### Detection of cell viability, changes related to apoptosis and DNA fragmentation analysis

**Figure 3:**
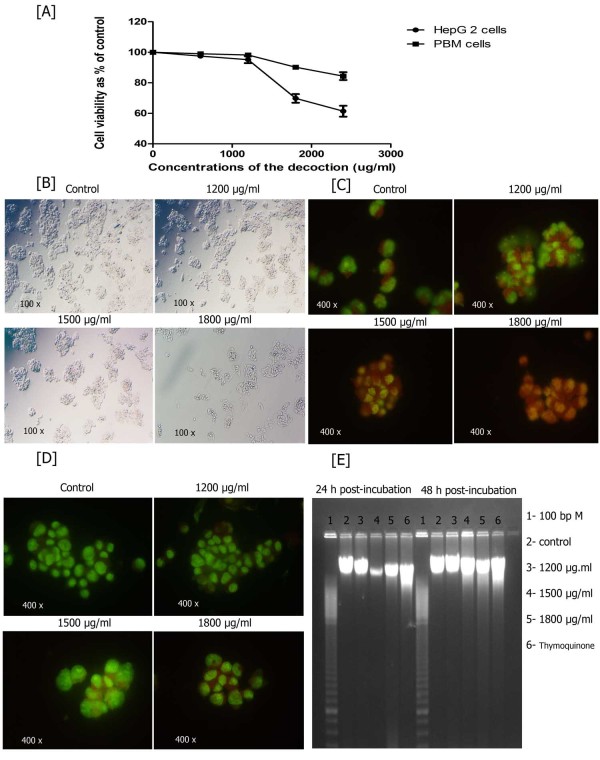
**Detection of morphological changes related to apoptosis and DNA fragmentation analysis**. Data are representative one of three similar experiments. Induction of decoction on morphological changes, apoptotic bodies formation and DNA fragmentation in HepG2 cells. The cells were treated with indicated dose of decoction for 24 h and (A) anti-proliferative effect on PBM cells and HepG2 cells (B) photographed by phase contrast microscope. (C) The cells were stained with acridine orange/ethidium bromide and visualized for nuclear morphology and apoptotic bodies under fluorescent microscope using a blue filter (D) The cells were stained with Hoechst 33258 and visualized for nuclear morphology and apoptotic bodies under fluorescent microscope using a blue filter (E) Fragmented DNA was extracted and analyzed on 2% agarose gel electrophoresis.

Effects of the decoction on cell viability in HepG2 cells and peripheral blood mononuclear cells were compared. As evident from Figure 3A, less anti-proliferative effect on normal PBM cells was observed when compared to the effect of the decoction in HepG2 cells. Characteristic apoptotic morphological changes of HepG2 cells were assessed by the light and fluorescent microcopy (Figure 3B and 3C). Fluorescent microscopic examination of HepG2 cells after AO/EB staining (Figure 3C) revealed nuclear condensation, membrane blebbing, nuclear fragmentation and apoptotic bodies (the hallmarks of apoptosis) in cells that had been incubated with the decoction. The number of cells with orange to red nuclear staining increased with increase in the dose of decoction. The control cells did not exhibit any of the above morphological changes. Chromatin condensation and other apoptotic characters were observed only in the treated cells and not in the control cells after staining with Hoechst 33258 (Figure 3D). DNA fragmentation (ladder pattern) characteristic of apoptosis was observed in HepG2 cells (2 × 10^5 ^cells/ml) exposed to the standardized decoction for 24 and 48 h. Control cells not exposed to the decoction, showed no evidence of DNA fragmentation (Figure 3E).

### Effect of the decoction on the expression of caspase 3 and caspase 9 in HepG2 cells

**Figure 4:**
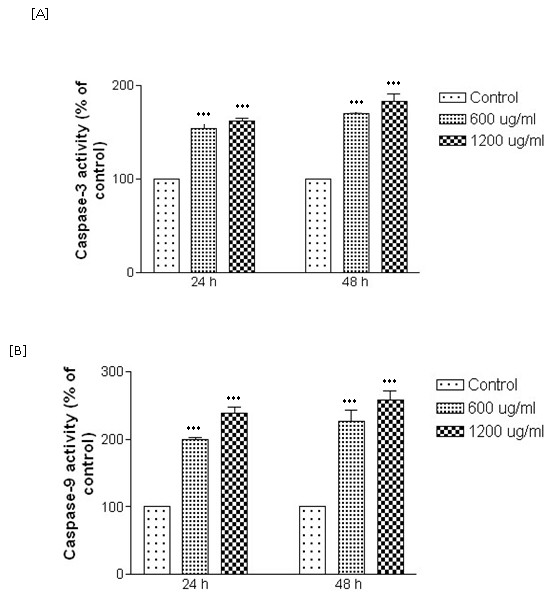
**Expression of (A) caspase-3 and (B) caspase-9 in HepG2 cells treated with the decoction**. Results are expressed as mean ± SEM of triplicate determinations (***p < 0.001 when compared to the control).

As shown in Figure 4 decoction increased the activation of caspase-3 and caspase-9 in HepG2 cells at concentration of 600 μg/ml and 1200 μg/ml in a significant (*p *< 0.001) time and dose dependent manner.

## Discussion

Previous in vivo and in vitro investigations [1,2] have shown that the decoction, comprised of *N. sativa *seed, *H. indicus *root, and *S. glabra*, can mediate anti-hepatocarcinogenic effects, without producing any significant toxic side effects. The antihepatocarcinogenic effects of the decoction probably results from an impact of this polyherbal formulation on a broad range of biochemical activities including antioxidant activity [5], anti-inflammatory activity, immunomodulatory activity and modulation of activities of factors involved in cell proliferation and cell cycle arrest [3,4,6]. In the study carried out by Thabrew *et al.*, (2005), a flow cytometric analysis of HepG2 cells exposed to the test decoction indicated that this polyherbal formulation also has the potential to induce apoptosis.

Results obtained in the present investigation confirm that apoptosis could indeed be a major mechanism through which the decoction mediates its anti-hepatocarcinogenic effect. Typical features of apoptosis such as chromatin condensation, and nuclear fragmentation were clearly evident in decoction treated HepG2 cells from light microscopic and fluorescent microscopic studies and DNA fragmentation analysis by agarose gel electrophoresis.

Two major pathways (the extrinsic and intrinsic pathways) regulate apoptosis. Both pathways converge to a common pathway involving the activation of a cascade of proteases named caspases that cleave regulatory and structural molecules, culminating in cell death. Caspases are the central components of the apoptotic response and a conserved family of enzymes that irreversibly commit a cell to die. They are cysteine proteases that cleave substrates after specific aspartate residues. The apoptotic caspases which are involved in the regulation and execution of apoptosis are generally divided into two classes: the initiator caspases, which include caspase- 2, -8, -9 and -10 and the effector caspases, which include caspases-3, -6 and -7 [18,19].

Due to differences in sensitivity, tumours are considered to arise more frequently through the intrinsic pathway than the extrinsic pathway [20]. Transmembrane receptor -mediated interactions consisting of death receptors (such as Fas), which are members of the tumour necrosis factor (TNF) receptor gene superfamily [21], are reported to be involved in initiation of apoptosis by the extrinsic pathway. In contrast, initiation of apoptosis by the intrinsic pathway involves a diverse array of non-receptor mediated stimuli. These stimuli produce intracellular signals that act directly on targets within the cell and are mitochondrial initiated events [22]. The release of mitochondrial proteins such as cytochrome c, promotes the assembly of a caspase - activating complex (the apoptosome) which in turn can induce activation of caspase-9 and thereby initiate the caspase cascades participating in apoptosis [23,24]. Caspase -9, the initiator protein, is considered to be an essential element in the intrinsic pathway of apoptosis [23]. Caspase - 3, is reported to be the promoter of apoptosis. Results of the present study demonstrate that the decoction under investigation can significantly enhance the activities of both caspase-3 and caspase-9 in HepG2 cells in a time and dose dependent manner.

It is well known that apoptosis is tightly regulated by changes in the expression of specific genes. Fas and Bcl2 families of genes are the best known among the factors that modulate cancer related apoptosis. Fas binding to its ligand FasL results in the initiation of active caspase-8 that can activate caspase-3 and induce cell apoptosis [25]. The Bcl-2 family of proteins is divided into three subfamilies and pro-apoptotic and anti-apoptotic members have a major role in the regulation of cell death. The anti-apoptotic subfamily comprises of Bcl-2, Bcl-xL, Bcl-w, Mcl-1, A1 (Bfl-1), and Bcl-B proteins and pro apoptotic subfamily consists of Bax, Bak, Mtd (Bok), and Bcl rambo. Anti-apoptotic members of the Bcl-2 family (among which Bcl-2 is of particular significance), inhibit the release of these apoptogenic factors, whereas pro-apoptotic members such as Bax, promote its release [26]. Both Bax and Bcl-2 play pivotal roles in caspase activation and the regulation of apoptosis [27,28]. In the present study we have demonstrated that in HepG2 cells, in response to the decoction comprising of *N. sativa *seed, *H. indicus *root, and *S. glabra*, transcription (as demonstrated by RT-PCR) and translation (as demonstrated by immunohistochemistry and western blot analysis) of pro apoptotic Bax gene are significantly enhanced while the transcription and translation of anti apoptotic Bcl-2 gene are significantly reduced.

Overall results obtained indicate that apoptosis is a probable mechanism through which the decoction under test mediates its anti-hepatocarcinogenic effect. To determine the actual percentage of apoptosis a FACS analysis using Annexin- V (FTC conjugated) needs to be conducted. Apoptosis induced by the decoction may be regulated by pro-apoptotic and anti-apoptotic protein members of the Bcl-2 family and is executed through two major caspase pathways, the mitochondria-dependent "intrinsic" cytochrome c/caspase- 9 and caspase-3 pathway.

## Conclusions

In conclusion, the present study demonstrates that the decoction of *Nigella sativa *seeds, *Hemidesmus indicus *roots and *Smilax glabra *rhizomes can induce apoptosis in human hepatocellular carcinoma HepG2 cell, in a dose and time dependent manner through the activation of caspase-3 and caspase-9, and up regulation of pro-apoptotic Bax and down regulation of anti-apoptotic Bcl-2 genes which are involved in intrinsic or mitochondrial pathway of apoptosis.

## Competing interests

The authors declare that they have no competing interests.

## Authors' contributions

SRS participated in design of the study, preparation of the manuscript and carried out all the experiments and edited the manuscript. IT participated in design of the study and preparation of the manuscript and edited the manuscript. PBG helped for the western blot analysis and literature search. KHT participated in the preparation of the manuscript and edited the manuscript. All authors read and approved the final manuscript.

## Pre-publication history

The pre-publication history for this paper can be accessed here:

http://www.biomedcentral.com/1472-6882/12/25/prepub

## Supplementary Material

Additional file 1 **Figure S1 Effects of the decoction on mRNA expression of Bax and Bcl-2 in HepG2 cells treated with the decoction for 12, 24, and 48 h**. Click here for file
