# Supplementary material for: Modulation of apoptosis in human hepatocellular carcinoma (HepG2 cells) by a standardized herbal decoction of Nigella sativa seeds, Hemidesmus indicus roots and Smilax glabra rhizomes with anti- hepatocarcinogenic effects
Source: BMC Complement Altern Med. 2012 Mar 29;12:25. doi: 10.1186/1472-6882-12-25 (PMC3364896; doi:10.1186/1472-6882-12-25)
Supplement: Additional file 1 — Figure S1 Effects of the decoction on mRNA expression of Bax and Bcl-2 in HepG2 cells treated with the decoction for 12, 24, and 48 h. [file 1472-6882-12-25-S1.DOCX]

**Supplementary Figure.1**. **Effects of the decoction on mRNA expression of Bax and Bcl-2 in HepG2 cells treated with the decoction for 12, 24, and 48h**
